# Correction: Trajectory analyses in insurance medicine studies: Examples and key methodological aspects and pitfalls

**DOI:** 10.1371/journal.pone.0315775

**Published:** 2024-12-10

**Authors:** Laura Serra, Kristin Farrants, Kristina Alexanderson, Mónica Ubalde, Tea Lallukka

## Notice of Republication

An incorrect version of Fig 1 was published in error. This article was republished on November 22, 2024 to correct for this error. Please download this article again to view the correct version.
