# Supplementary material for: Trajectory analyses in insurance medicine studies: Examples and key methodological aspects and pitfalls
Source: PLoS One. 2022 Feb 11;17(2):e0263810. doi: 10.1371/journal.pone.0263810 (PMC8836370; doi:10.1371/journal.pone.0263810)
Supplement: Attachment — Submitted filename: Response to Editor_data availability.pdf [file pone.0263810.s001.pdf]

1. Please clarify how future researchers can request access to the data underlying your study. Please provide any relevant information that may be helpful in accessing the data (such as contact information & data set names)

The data that support the findings of this study come, on the one hand, from an annual sample of Spanish Social Security affiliates that are part of the Continuous Working Life Sample (MCVL by its acronym in Spanish), provided by the General Directorate of Social Security (DGOSS by its acronym in Spanish) and, on the other hand, from the linked records of sickness absence episodes certified in Catalonia provided by Catalan Institute for Medical and Health Evaluations (ICAM by its acronym in Catalan).

Future researchers should contact these two public institutions in order to request access to the data underlying this study as they are not publicly available. To access those sensitive data, as in most countries, ethical permission from the researcher's institution and the respective data holding authorities is needed. All researchers granted such permissions can access these type of data.

The MCVL data can be obtained filling the PDF form available online at https://www.seg-social.es/wps/portal/wss/internet/EstadisticasPresupuestosEstudios/Estadisticas/EST211/1459 and sending it to the DGOSS (mcvl.dgoss-sscc@seg-social.es) after having acquired ethical permission for handling these type of sensitive data. These data files are generated annually and DVDs with older versions of the MCVL can be requested. The versions from 2012 to 2014 were used for this study.

Access to linked records of sickness absence episodes provided by the ICAM was possible thanks to an agreement signed between DGOSS, ICAM and CiSAL-UPF. This records linkage is made under strict confidentiality control and is done directly by DGOSS and ICAM. The resulting pseudonymised data can be requested by contacting the ICAM (bustia.icam@gencat.cat) and reaching an agreement to obtain the sickness absence data (ITCC by its acronym in Catalan) from 2012 to 2014.

2. Please confirm the authors had no special access privileges to the data others would not have.

Authors of this study had no special access privileges to the data and other researchers would obtain the same information after being granted their access by the DGOSS and the ICAM.
